# Application of Clavien–Dindo classfication-grade in evaluating overall efficacy of laparoscopic pancreaticoduodenectomy

**DOI:** 10.3389/fsurg.2023.1043329

**Published:** 2023-03-03

**Authors:** Xiangyang Song, Yu Ma, Hongyun Shi, Yahui Liu

**Affiliations:** Department of Hepatobiliary and Pancreatic Surgery, General Surgery Center, The First Hospital of Jilin University, Changchun, China

**Keywords:** Clavien–Dindo classification, complications, surgery, laparscope, pancreaticoduodenectomy

## Abstract

**Background:**

The Clavien–Dindo classification (CDC) has been widely accepted and applied in clinical practice. We investigated its effectiveness in prediction of major complications (LPPC) after laparoscopic pancreaticoduodenectomy (LPD) and associated risk factors.

**Methods:**

A retrospective analysis was conducted covering clinical data of 793 patients undergoing LPD from April 2015 to November 2021. CDC was utilized to grade postoperative complications and analyze the differences. Risk factors of LPPC were identified according to univariate and multivariate analyses.

**Resluts:**

For the 793 patients undergoing laparoscopic pancreaticoduodenectomy in the northeast of China, LPPC was reported in 260 (32.8%) patients, pancreatic fistula in 169 (21.3%), biliary fistula in 44 (5.5%), delayed gastric emptying in 17(2.1%), post pancreatectomy hemorrhage in 55 (6.9%), intestinal fistula in 7 (0.8%), abdominal infections in 59 (7.4%) and pulmonary complication in 28 (3.5%). All complications were classified into five levels with the C–D classification (Grade I–V), with 83 (31.9%) patients as grade I, 91 (35.0%) as grade II, 38 (14.6%) as grade IIIa, 24 (9.2%) as grade IIIb, 9 (3.5%) as grade IV and 15 (5.8%) as grade V. 86 (10.8%) patients experienced major complications (grade III–V).The results of univariate and multivariate analysis revealed the independent risk factors for laparoscopic pancreaticoduodenectomy complications to be preoperative total bilirubin (*P* = 0.029, OR = 1.523), soft pancreas texture (*P* < 0.001, OR = 1.399), male (*P* = 0.038, OR = 1.396) and intraoperative transfusion (*P* = 0.033, OR = 1.517). Preoperative total bilirubin (*P* = 0.036, OR = 1.906) and intraoperative transfusions (*P* = 0.004, OR = 2.123) were independently associated with major postoperative complications. The influence of different bilirubin levels on C–D grade of complications was statistically significant (*P* = 0.036, OR = 1.906).

**Conclusions:**

The Clavien–Dindo classification (CDC) may serve as a valid tool to predict major postoperative complications and contribute to perioperative management and comparison of surgical techniques in different medical centers.

## Introduction

Pancreaticoduodenectomy (PD), as a preferred treatment for malignant diseases of the head of pancreas, distal bile duct and periampullary. In contrast, greater advantages have been reported of laparoscopic surgery over PD (1–3). Despite the significant modifications in medical technology, the complication rates are still reported to be around 50% (4–7) in high volume centers, which has prolonged hospital stays, bringing mental burden to patients and aggravate health care costs. Hence, the overall evaluation of surgical complications has absorbed great concern in recent years.

Over the past decade, there have appeared various definitions of postoperative complication. For instance, the international study group of pancreatic surgery (ISGPS) reported a definition of post pancreatectomy hemorrhage (PPH) (8) and postoperative pancreatic fistula(POPF) (9). Charles J (10) defined the delayed gastric emptying (DGE) which requires postoperative nasogastric tube decompression for over 10 days. However, these definitions are only rooted in a single system, without the available established criteria to standardize surgical complications. The lack of a uniform criterion involving all systemic complications impedes effective comparison of surgical outcomes and levels of practice across medical bases, resulting in inaccurate recording of major complications incidence.

In terms of the categorization of postoperative complications of LPD, ISGPS has introduced a series of definitions, which have received wide adoption and favor from domestic and international surgical groups. However, these definitions are limited to only a specific class of PPC, covering PPH, POPF and DGE, and show the unique gas assessment criteria for a specific complication, which requires the assessment and exploration on the relevant risk factors only for a specific class of complication, while inefficient for the synergy and risk factors among these different classifications of complications. Secondly, a simple, reproducible evaluation that works for all types of postoperative complications is required considering the increasing health care need and medical costs, the limited resources, and variation in clinical perioperative data, so as to achieve the long-term comparisons between medical centers, between surgical modalities, and within the same center. The C–D grading system developed by Clavien et al. provides such a new approach. Dindo proposed (11) an modified grading system referring to complication management in 2004, which has been widely adopted by surgeons around the world. The Japan Clinical Oncology Group (12) set up a committee and detailed the grading criteria based on the rules of CDC. Laura (13) utilized CDC to explore the impact of complications following minimally invasive esophagectomy on survival. Dong-Kyu (14) also evaluated complications after small bowel resection depending on CDC. While limited was known about the application of CDC to LPD. The objective of this study is to identify risk factors for LPPC and to determine their association with CDC through a retrospective analysis of the largest LPD volume center in northeast China. By evaluating the overall postoperative efficacy of LPD, we hoped to make a contribution to a personalized management of patients undergoing LPD.

## Patients and methods

All patients who underwent LPD at the First Affiliated Hospital of Jilin University from April 2015 to November 2021 were involved in this study, which was approved by the hospital. A prospective electronic database was maintained to provide all the data, containing all of the patients' outpatient and inpatient information, covering preoperative laboratory parameters (serum total bilirubin), preoperative biliary drainage, common disease(hypertension, diabetes, hepatitis), patients characteristics, preoperative surgical factors, outcomes and postoperative treatment. Considering the varying views of different surgeons on the indications for surgery, the serum albumin and hemoglobin were maintained above 35 g/L and 100 g/L, respectively, before surgery here. Therefore, these two variables were excluded from the study. The patient had signed an informed consent for the data to be used in the clinical study. The information will be maintained strictly confidential. The study was approved by the First Affiliated Hospital of Jilin University and all methods were performed in accordance with the relevant guidelines and regulations.

Patients with these identifications were not included in the model. First, preoperative enhanced computerized tomography (CT) or magnetic resonance imaging (MRI) indicate distant metastasis of malignant cells. Second, intraoperative tumors invade arteries, veins and surrounding vital organs, or extensive abdominal metastasis fails to be completely resected. Third, due to bleeding or severe tissue adhesion, intraoperative tumors are difficult to operate and switch to open.

## Surgery

All procedures following the standard of classical Whipple surgery were performed by four experienced surgeons through minimally invasive laparoscopy. Removed organs referred to the gastric pylorus, distal antrum of the stomach, duodenum, cholecyst, distal common bile duct, proximal jejunum and head of pancreas. The gastric antrum and neck of the pancreas were disconnected by endovascular gastrointestinal anastomosis stapler, without performing enlarged lymph node dissection. Digestive tract reconstruction was performed by Child method. Pancreatoenteric was performed by means of pancreatic duct anastomosis to jejunum mucosa. The remaining pancreas was routinely placed with a supportive tube to ensure the smooth drainage of pancreatic fluid. Abdominal drainage tubes were indwelled in front and rear of pancreaticoenteric anastomosis and around bilioenteric anastomosis. All patients received cefoperazone shock therapy before surgery, routine prophylactic therapy with antibiotics (Cefoperazone 1 g, BID, intravenous drip) and somatostatin (Stilamin 6 mg, QD, intravenous drip) after surgery, given hemostatic drugs to prevent bleeding.

## Complications

Case records were reviewed for each enrolled patient to identify complications, including PPH, POPF, DGE, biliary fistula, abdominal infections, pulmonary complication, and intestinal fistula. PPH,POPF,DGE and biliary fistula were defined in ISGPS standards (8, 9, 15, 16). All complications were graded (grade I–V) per Clavien–Dindo classification. Major complications were defined as severely greater than or equal to grade III. Mortality was defined as death within 30 days after surgery or during hospitalization. Mortality is the rate of grade V complications.

The specific grading criteria are: (1) Grade I: Any deviation from the normal postoperative normal recovery process that includes only the use of antiemetics, antipyretics, analgesics without requirement of pharmacological treatment, surgical intervention, endoscopic or interventional treatment. Only those can be resolved with antiemetics, antipyretics, analgesics, diuretics, rehydration and physical chemotherapy are included, as well as the infected wounds that can be managed at the bedside. (2) Grade II: Complications requiring medications in addition to those listed in Class I. Blood transfusion and total parenteral nutrition are also included. (3) Grade III: Complications requiring surgical intervention, intervention, endoscopic treatment, and total parenteral nutrition. Those require general anesthesia are categorized into level IIIa, and those do not into level IIIb. (4) Level IV: Life-threatening complications (including central nervous system complications) that require intensive care unit treatment, with single-organ failure at level IVa (including the need for dialysis) and multi-organ failure at level IVb. (5) Grade V: death.

## Statistical analysis

Normally distributed measurement data were represented by mean and standard deviation, with difference compared by Student's *t*-tests. Non-normally distributed continuous variables were reported as the median with interquartile range and were compared by Mann–Whitney *U*-tests, with categorical variables compared by *Χ*^2^ test or Fisher's exact test. Univariate analysis covered all potential indicators, including preoperative, intraoperative and postoperative patient-related factors. Multivariate logistic regression analysis including the potential factors with *P* ≤ 0.05 in univariate analysis was conducted to identify the risk factors associated with all and major complications after LPD. Potential interactions between these factors and the level of complications were also examined. Results were represented by *P*-values, odd ratios(ORs)and 95% confidence intervals (CIs). *P*-value of ≤0.05 was considered statistically significant difference. All statistic analyses were performed using software SPSS Version 25.0.

## Results

### Cohort basic characteristics

From April 2015 to November 2021,824 patients underwent LPD at the First Affiliated Hospital of JiLin University. 31 patients were excluded owing to the lack of data. The basic characteristic and surgical details of the patients were listed in Table 1. The median age of 793 patients was 60 (IQR: 52–66) years, composed of 442 (55.7%) males and 351 (44.3%) females. Mean total bilirubin was 94.23 ± 85.67 mmol/L. Patients with mean cancer antengin19-9 of 154.05 ± 197.59 U/I.321 (40.5%) underwent ultrasonic-guided bile drainage due to hyperbilirubinemia before surgery.112 (14.1%) had hypertension, 103 (13.0%) had diabetes and 25 (3.2%) had virus hepatitis. 34(4.3%) patients were classified as ASA I, 640 (80.7%) as ASA II and 119 (15.0%) as ASA III. Median blood loss was 50 (20–100) ml. Mean operation time was 191.02 ± 66.90 min. Pancreatic specimens were soft in 424 (53.5%) patients, middle in 135 (17.0%) patients and firm in 234 (29.5%) patients. 408 (51.5%) patients was found to exhibit pancreatic duct diameter ≤ 3 mm 385 (48.5%) patients found >3 mm. Other baseline characteristics, intraoperative details are described in Table 1.

**Table 1:** Baseline characteristics of the study cohort.

Variables	Value
Total	793 (100)
Sex (female/male, *n*%)	351 (44.3)/442 (55.7)
Age (years, IQR)	60 (52–66)
BMI (kg/m^2^, SD)	22.97 ± 3.23
Preoperative CA19-9 (U/I, SD)	154.05 ± 197.59
Preoperative TBIL (mmol/L,SD)	94.23 ± 85.67
Hypertension (yes/no, *n*%)	112 (14.1)/681 (85.9)
Diabetes (yes/no, *n*%)	103 (13.0)/690 (87.0)
Virus hepatitis (yes/no, *n*%)	25 (3.2)/768 (96.8)
Preoperative biliary drainage (yes/no, *n*%)	321 (40.5)/472 (59.5)
ASA grade (I/II/III, *n*%)	34 (4.3)/640 (80.7)/119 (15.0)
History of abdominal surgery (yes/no, *n*%)	139 (17.5)/654 (82.5)
Vascular variation (yes/no, *n*%)	560 (70.6)/233 (29.4)
Introperative bleeding (ml, IQR)	50 (20–100)
Intraoperative transfusions (yes/no, *n*%)	145 (18.3)/648 (81.7)
Operation time (min, SD)	191.02 ± 66.90
Pancreas texture (firm/middle/soft, *n*%)	234 (29.5)/135 (17.0)/424 (53.5)
Size of pancreatic duct (>3/≤3, *n*%)	385 (48.5)/408 (51.5)

BMI, body mass index; IQR, interquartile range; SD, mean; CA19-9, cancer antigen 19-9, TBIL, total bilirubin; ASA, American Society of Anesthesiologists.

LPPC occurred in 260 (32.8%) patients, with 169 (21.3%) patients developing POPF, 44 (5.5%)patients developing biliary fistula, 17 (2.1%) patients developing DGE, 55 (6.9%) patients developing PPH, 7 (0.8%) patients developing intestinal fistula, 59 (7.4%) patients developing abdominal infections and 28 (3.5%) patients developing pulmonary complication. According to CDC, the LPPC of all patients could be divided into five grades (Grade I–V), of which grade III was subdivided into grade IIIa and grade IIIb according to whether there was invasive operation under general anesthesia. POPF was determined to be the most common complication after LPD in our study. In Table 2 the detailed classification of complications is shown. The number of patients with C–D grade I, II, IIIa, IIIb, IV and V was 83 (31.9%), 91 (35.0%), 38 (14.6%), 24 (9.2%), 9 (3.5%) and 15 (5.8%). The grade I–II was classified as mild LPPC and grade III–V as severe LPPC. 174 (66.9%) patients were categorized with grade I–II and 86 (33.1%) with grade III–V. The 793 patients were further divided into two groups: 707 (89.2%) patients with no or mild LPPC, and 86 (10.8%) patients with severe LPPC, among which 15 (1.9%) patients experienced postoperative death, 6 (40.0%) died of multiple organ failure due to severe postoperative infection, 4 (26.6%) died due to abdominal bleeding and failure in stopping bleeding after secondary laparotomy, 3 (20.0%) died of respiratory failure, 1 (6.7%) died two weeks after discharge with a large amount of blood visible in the abdominal drainage tube, which was considered to be arterial stump bleeding, and 1 (6.7%) died of pulmonary embolism.

**Table 2:** Clavien–Dindo classification of postoperative complications.

Complications	Total	Grade I	Grade II	Grade IIIa	Grade IIIb	Grade IV	Grade V
Pancreatic fistula	169	60	61	30	3	6	9
Hemorrhage	55	1	2	8	24	2	7
Delayed gastric emptying	17	1	8	3	1	4	0
Billary fistula	44	13	12	14	2	1	2
Abdominal infections	59	6	15	18	7	7	6
Pulmonary complication	28	8	7	5	1	2	5
Intestinal fistula	7	0	1	3	1	1	1

### Risk factors of LPPC

The results of univariate analysis of postoperative complications and severe complications were listed in Table 3 and those of multivariate analysis in Table 4. In univariate analysis, gender (*P* = 0.006), soft pancreatic texture (*P* < 0.001) and pancreatic duct diameter ≤ 3 mm (*P* = 0.009) were significantly associated with LPPC, while BMI (*P* = 0.027), preoperative total bilirubin (*P* = 0.010), preoperative biliary drainage (*P* = 0.049) and intraoperative blood transfusion (*P* = 0.015) were associated with LPPC. Severe LPPC was significantly associated with preoperative TBIL > 170 mmol/L (*P* < 0.001) and intraoperative blood transfusion (*P* = 0.002), appearing to be related with size of pancreatic duct (*P* = 0.045).

**Table 3:** Univariate analysis of postoperative complications and severe complications.

		No-LPPC *n* = 533	LPPC *n* = 260	*P*-value	Grade 0–II *n* = 707	Grade III–V *n* = 86	*P*-value
Age	<65/≥65	365/168	178/82	0.996	490/217	53/33	0.148
Sex	Female/male	254/279	97/163	0.006**	315/392	36/50	0.635
BMI (kg/m^2^)	≤23.9/>23.9	366/167	158/102	0.027*	471/236	53/33	0.356
CA19-9 (U/I)	≤100/>100	302/231	165/95	0.068	421/286	46/40	0.281
Vascular variation	yes/no	153/380	80/180	0.549	209/498	24/62	0.750
Preoperative TBIL (mmol/L)	≤170/>170	448/85	199/61	0.010**	589/118	58/28	0.000**
Preoperative biliary drainage	Yes/no	203/330	118/142	0.049*	279/428	42/44	0.094
Hypertension	Yes/no	77/456	35/225	0.708	100/607	12/74	0.962
Diabetes	Yes/no	65/468	38/222	0.341	91/618	12/74	0.778
Virus hepatitis	Yes/no	13/520	12/248	0.100	22/685	3/83	0.850
History of abdominal surgery	Yes/no	92/441	47/213	0.777	121/586	18/68	0.380
ASA grade	≤II/>II	454/79	220/40	0.835	601/106	73/13	0.976
Operation time (min)	≤300/>300	492/41	242/18	0.698	658/49	76/10	0.117
Pancreas texture	Firm/middle/soft	174/100/259	60/35/165	0.000*	373/120/214	51/15/20	0.386
Size of pancreatic duct	>3/≤3	276/257	109/151	0.009**	352/355	33/53	0.045*
Introperative bleeding (ml)	≤400/>400	506/27	241/19	0.205	668/39	79/7	0.326
Intraoperative transfusions	Yes/no	85/448	60/200	0.015*	119/588	26/60	0.002**

BMI, body mass index; CA19-9, cancer antigen 19-9, TBIL, total bilirubin; ASA, American Society of Anesthesiologists. Grade 0–II, no complications and Clavien–Dindo classification grade I–II.

* *P* < 0.05.

** *P* < 0.01.

**Table 4:** Multivariate analysis of postoperative complications and severe complications.

		OR-value	95%confidence interval		*P*-value
			Lower	Upper	
**No-LPPC/LPPC**					
Pancreas texture	Firm/Middle/Soft	1.399	1.170	1.673	0.000
Intraoperative transfusions	no	1			
	yes	1.517	1.034	2.226	0.033
sex	Female	1			
	Male	1.396	1.019	1.911	0.038
Preoperative TBIL (mmol/L)	≤170	1			
	>170	1.523	1.043	2.224	0.029
**Grade 0–II/III–V**					
Preoperative TBIL (mmol/L)	≤170	1			
	>170	2.313	1.406	3.807	0.001
Intraoperative transfusions	否	1			
	是	2.123	1.278	3.529	0.004

LPPC, post-laparoscopic pancreaticoduodenectomy complications; TBIL, total bilirubin; Grade 0–II, no complications and Clavien–Dindo classification grade I–II.

In multivariate Logistic regression analysis, pancreatic texture (*P* < 0.001, OR = 1.399, 95% CI: 1.170–1.673), intraoperative blood transfusion (*P* = 0.033, OR = 1.517, 95% CI, 1.034–2.226), gender (*P* = 0.038, OR = 1.396, 95% CI: 1.019–1.911) and preoperative TBIL > 170 mmol/L (*P* = 0.029, OR = 1.523, 95% CI: 1.043–2.224) were independent risk factors for postoperative complications of LPD. Severe LPPC was revealed to be independently associated with preoperative TBIL > 170 mmol/L (*P* = 0.001, OR = 2.313, 95% CI: 1.406–3.807) and intraoperative transfusion (*P* = 0.004, OR = 2.123, 95% CI: 1.278–3.529).

Analysis of differences between mild and severe complications.

As shown in Table 5, in comparison with mild complications (grade I–II), severe complications (grade III–V) were associated with preoperative CA19-9 (*P* = 0.019), preoperative TBIL > 170 mmol/L (*P* = 0.015), and total operation time (*P* = 0.036). Multivariate analysis suggested preoperative TBIL > 170 mmol/L (*P* = 0.036, OR = 1.901, 95% CI: 1.043–3.484) as an independent risk factor.

**Table 5:** Univariate and multivariate analysis of complication grading.

	Univariate				Multivariate			
		Grade I–II *n* = 174	Grade III–V *n* = 86	*P*-value	OR-value	95%CI		*P*-value
						Lower	upper	
Age	<65/≥65	125/49	53/33	0.095				
Sex	Female/male	61/113	36/50	0.286				
BMI (kg/m^2^)	≤23.9/>23.9	105/69	53/33	0.842				
CA19-9 (U/I)	≤100/>100	119/55	46/40	0.019*				
Vascular variation	Yes/no	56/118	24/62	0.482				
Preoperative TBIL (mmol/L)	≤170/>170	141/33	58/28	0.015*	1.906	1.043	3.484	0.036*
Preoperative biliary drainage	Yes/no	76/98	42/44	0.432				
Hypertension	Yes/no	23/151	12/74	0.870				
Diabetes	Yes/no	26/148	12/74	0.832				
Virus hepatitis	Yes/no	9/165	3/83	0.543				
History of abdominal surgery	Yes/no	29/145	18/68	0.401				
ASA grade	≤II/>II	147/27	73/13	0.933				
Operation time (min)	≤300/>300	166/8	76/10	0.036*				
Pancreas texture	Firm/middle/soft	40/20/114	20/15/51	0.394				
Size of pancreatic duct	>3/≤3	76/98	33/53	0.415				
Introperative bleeding (ml)	≤400/>400	162/12	79/7	0.717				
Intraoperative transfusions	Yes/no	34/140	26/60	0.054				

BMI, body mass index;CA19-9,cancer antigen 19-9,TBIL,total bilirubin;ASA, American Society of Anesthesiologists.

* *P* < 0.05.

**Figure 1:**
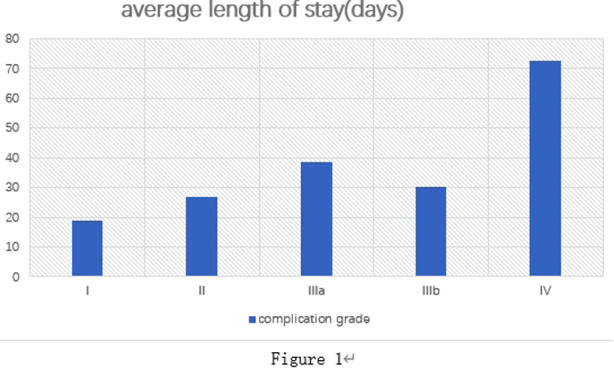
Length of stay for complications with different C-D grades.

The hospital stay of patients with complications of all grades was evaluated as grade I (19.06 ± 4.575), II (26.82 ± 6.251), IIIa (38.66 ± 9.737), IIIb (30.33 ± 12.815), IV (72.78 ± 10.721) and V (22.60 ± 11.564). As depicted in Figure 1, except for patients who died, the length of postoperative hospital stay was generally prolonged with the elevation of the LPPC grade.

## Discussion

PD is the primary choice in the treatment of periampullary tumor, which has even become a representative of advanced surgery celebrated by its high degree of difficulty. In recent years, laparoscopy has been favored by surgeons with its advantages of small trauma, low pain and quick recovery, as minimally invasive Whipple has been widely carried out in general surgery around the world (17–21). The service of this operation involves a number of organs and makes great impacts in human anatomy. Despite the modification of technique and clinical nursing level in recent years, its complication rate is still reported as high as 50%–60% (22, 23). In this study, the complications rate was reported to be only 32.8% (260/793). There has always existed a competitive relationship between the global general surgery and the medical center in completing LPD with a low postoperative mortality rate, which will undoubtedly win a better public praise and reputation, leading the forefront of surgery at home and abroad. Therefore, the existence of a unified standard to compare the efficacy of LPD in different regions and countries is required, and the C–D classification provides such a tool, which as a grading standard has been referred to in many surgical fields (24–26).

The C–D grading itself has several advantages (11). First, distinguished from the traditional single-system study, it evaluates the surgical efficacy from the overall multi-system. Secondly, it can prepare for the assessment of potential independent risk factors for surgery-related complications. Finally, it can contribute to exploring the factors that may aggravate the complications, thus fundamentally reducing the occurrence of such events, which benefits the surgical field as a whole.

This study concluded that soft pancreas could serve as an independent risk factor for postoperative complications of LPD, which is also consistent with the view of most scholars. The soft pancreas (26, 27) generally has a good exocrine function with the capability to secrete a large amount of pancreatic fluid. During pancreatic jejunal anastomosis after LPD, it is easy to corrode the anastomotic vessels and tissues. Secondly, when the soft pancreas is anastomosed with the residual pancreas, cutting effect is more likely appear by the suture and lead to pancreatic damage, which will cause POPF, resulting in bleeding, abdominal infection, sepsis and other complications, which has reached a consensus in academic (27–30). The pancreas with low density is more sensitive to inflammation compared to those with high fibrosis. With the subside of inflammation after surgery, the volume of the remaining pancreas will be slightly reduced, and the gap between the suture and the tissue will also develop, which also provides an opportunity for pancreas fluid leakage.

The diameter of pancreatic duct (5, 28, 29) is related to LPPC, which with excessively thin duct is associated to the higher occurrence of damage in the pancreas when anastomosing with jejunum mucosa, and difficult to exact anastomosis. Another study in our center (27) demonstrated the diameter of small pancreatic duct as an independent risk factor for postoperative POPF (OR: 30.277, 95% CI: 10.578–86.655, *P* < 0.001), which was also verified in other studies. However, the expanded sample size resulted in the statistically insignificant diameter of pancreatic duct in the multivariate analysis in the present study. We speculate the other LPPC resulting from pancreatic juice when POPF occurs after LPD, such as PPH, abdominal infection, etc., so the diameter of pancreatic duct is considered to be related to LPPC. However, due to the absence of uniform standard for the measurement of the diameter, which is thus estimated roughly according to the experience of the operator, these data may be biased, further verification from other medical centers is required.

In the study, male sex was a risk factor of LPPC, but exhibited no significant association. Most studies (31–33) have not reported that gender differences affect the rate of postoperative complications. We considered this result to be related to the living habits of people in northeast China. In northeastern China, table culture is a weighted means of communication, especially alcohol consumption, which is a main cause of chronic pancreatitis. Although the hard pancreas are almost universally accepted more likely to reduce the incidence of postoperative complications in terms of technique, some scholars (34, 35) argue that the excessive fibrosis of the pancreas can affect the development of pancreatic anastomosis stoma, tending to leave gaps between the pancreas and jejunum in the process of stitching, and possible lacuna between pancreatic duct and supporting tube, which will be the hidden trouble to the patient outcome. It is also believed that men and women have different fat distribution and patients with more abdominal fat also have more fat in the pancreas, which affects the texture of the pancreas (36–38) and produce a certain impact on prognosis. However, the effect of gender (39–41) or history of chronic pancreatic on LPD prognosis still requires further study due to lack of enrolled studies, which may have a strong regional character.

Despite the necessity of perioperative blood transfusion for patients with large blood loss during major surgery, it has been determined that blood transfusion is significantly associated with postoperative complications (42). We found that intraoperative blood transfusion was an independent risk factor for LPPC, possibly related to the systemic inflammatory response that blood transfusion may elicit after surgery. Large transfusions of red blood cells can also result in dilution clotting factors deficiency (43–46). Dirk J et al. (47) reported that the odds ratio for exposure to intraoperative blood transfusion in patients was 1.74. Some scholars (48) have concluded a linear correlation between blood transfusion and postoperative morbidity. The elevated risk of postoperative infection may be resulted from the immunosuppression caused by blood transfusion, which inhibits the activity of immune cells, such as T-cells and nature killer cells, and may promote the release of some growth factors, thus inducing tumor recurrence. Therefore, the indication of blood transfusion should be strictly grasped.

High bilirubin itself is a manifestation of liver damage. In surgery, cholestatic liver damage is often caused by biliary obstruction, which results in insufficient synthesis of coagulation factors and increased risk of postoperative bleeding (6, 49). There also have studies clearly reporting a higher incidence of liver failure or multiple organ failure in patients with high preoperative bilirubin levels (50–53). Vitamin K deficiency is common in patients undergoing preoperative bile drainage, which affects clotting factors synthesis, as well as in patients with obstructive liver injury. It has been suggested that mildly elevated bilirubin induced platelet activation *via* mechanism related to collagen-induced platelet activation, thereby inhibiting coagulation (54). All of these increase the risk of bleeding after surgery. This study suggests hyperbilirubinemia as an independent risk factor for postoperative complications of LPD, and is closely associated with the incidence of severe complications, which may even contribute to postoperative deterioration of the disease. One study (27) from a large capacity center in western China covering 1056 patients also identified hyperbilirubinemia as an independent risk factor for LPPC, especially highly correlated with Grade V (*P* = 0.042, 95% CI: 1.849 to 4.789, OR = 2.017). In univariate analysis, preoperative biliary drainage exhibited no statistical significance after excluding the interference of other factors after inclusion in regression model. Some scholars (55–57) believe that preoperative biliary drainage aggravates the risk of postoperative biliary tract infection, while it is undeniable that the alleviation of jaundice by preoperative drainage can significantly improve liver function with the potential to optimize the prognosis of patients (58). In multivariate analysis of this study, the *P*-values of bilirubin were 0.029, 0.001 and 0.036, respectively. There showed statistically significance between mild and severe complications (*P* = 0.001). Consideration of bilirubin not only increases the incidence of LPPC, but also may lead to the development of severe complications.

As shown in Figure 1, the hospital stay after surgery is generally extended with the improvement of LPPC level. Therefore, the C–D grading system is expected to improve perioperative patient management, shorten hospital stay, reduce medical costs and patient economic pressure.

In summary, we concluded the significant association of the results of CDC with risk factors for LPPC, which may accurately predict the major complications. This grading system could provide a reliable means of quality assessment in surgical procedures and contribute to date comparison among different medical bases and therapies. It may also be widely applied in abdominal surgery in the future. All of this will help modify the quality of minimally invasive surgery, contributing shorter hospital stay and decreased financial costs.

According to this study, we believe that the C–D system in clinical management can predict the postoperative recovery of patients. By analyzing the differences between complications of different severity, we found that certain factors such as hyperbilirubinemia and intraoperative blood transfusion were statistically significant, which suggests that we should pay more attention to the presence of such factors in patient management and try to correct preoperative hyperbilirubin as much as possible. We hope to establish a new scoring system. We can score by relevant preoperative risk factors, and then estimate the possibility of complications at all levels after surgery. However, the sample size of our center is limited, and we are unable to complete it for the time being. In addition, we are collecting new data. When the sample size is sufficient, we will further verify the results of this study and establish a new scoring system as far as possible. It is also hoped that other large capacity centers at home and abroad can further verify this experiment.

The study, as a single-center retrospective analysis, also has some limitations. First, the data were collected prospectively, possibly biasing in the process of information collection, and selection bias may exist in the selection of research objects. Second, the sample size is only concentrated in one region. Third, some variables were not considered in the study due to different treatment concepts. The results of this study require to be further verified by multi-center, accurately designed and reliable prospective studies in large-capacity centers to obtain more valuable results.

## Data Availability

The original contributions presented in the study are included in the article/Supplementary Material, further inquiries can be directed to the corresponding author.
